# Associations between local government expenditures and low birth weight incidence: Evidence from national birth records

**DOI:** 10.1016/j.pmedr.2019.100985

**Published:** 2019-08-30

**Authors:** David S. Curtis, Thomas E. Fuller-Rowell, Silvia Vilches, Joseph Vonasek, Nancy M. Wells

**Affiliations:** a Department of Family and Consumer Studies, University of Utah, Salt Lake City, UT 84112, United States of America; b Department of Human Development and Family Studies, Auburn University, Auburn, AL 36849, United States of America; c Department of Political Science, Auburn University, Auburn, AL 36849, United States of America; d Design + Environmental Analysis Department, Cornell University, Ithaca, NY 14853, United States of America

**Keywords:** Infant, low birth weight, Health status disparities, Parks, recreational, Housing, Community development

## Abstract

Local governments play an integral role in providing public services to their residents, yet the population health benefits are frequently overlooked, especially when services are outside the traditional health domain. With data from the U.S. Census of Governments and national birth records (spanning from 1992 to 2014), we examined whether local government expenditures on parks and recreation services (PRS) and housing and community development (HCD) predicted county low birth weight outcomes (population incidence and black-white disparities). Hypotheses were tested using bias-corrected county-by-period fixed effects models in a sample of 956 U.S. counties with a total of 3619 observations (observations were defined as three-year pooled estimates), representing 24 million births. Adjusting for prior county low birth weight incidence, levels of total operational, health, and hospital expenditures, and time-varying county sociodemographics, an increase in per capita county PRS expenditures of $50 was associated with 1.25 fewer low birth weight cases per 1000. Change in county HCD expenditures was not associated with low birth weight incidence, and, contrary to hypotheses, neither expenditure type was linked to county black-white disparities. Further examination of the benefits to birth outcomes from increasing parks and recreation services is warranted.

## Highlights

• Rising parks and recreation expenditures are linked to declining low birth weight.
• Housing and community expenditures are not associated with low birth weight.
• ‘Health’ was the only expenditure type linked to black-white perinatal disparities.
• Benefits of parks and recreation services may include healthier birth outcomes.

## 1 Introduction

The United States has high infant mortality relative to its economic peer countries, with black Americans bearing an especially large share of the burden (Heisler, 2012). The relative ranking of the U.S. in infant mortality and black-white disparities stem from disproportionate low birth weight and preterm birth rates (Heisler, 2012). To meet national goals of reducing adverse birth outcomes, innovative preventive health strategies are needed. County and municipal governments provide a range of services relevant to perinatal outcomes. Salient examples include health and hospital services (Bekemeier et al., 2014; Grembowski et al., 2010), yet other social programs may have a positive impact. Any service that improves women's physical or mental health or changes fertility patterns, for instance, could influence low birth weight (LBW) (Kramer et al., 2000; Giscombé and Lobel, 2005). Although extant literature is scarce on the maternal health benefits of many public services, increases in county government expenditures on housing and community development, parks and recreation services, and other social services are linked to improved county health outcomes (McCullough and Leider, 2016; Mueller et al., 2019a). Local government efforts to expand many public services, especially among county governments, is therefore noteworthy and highlights the need for research into health impacts (Benton et al., 2007; Kaczynski and Crompton, 2006; Lobao and Kraybill, 2005).

One health promotion strategy for local governments is to ensure public green spaces and opportunities for physical activity (Hunter et al., 2015; Wells et al., 2010). For example, increasing state government expenditures for parks and recreation services (PRS) are associated with greater physical activity and outdoor recreation (Cawley et al., 2007; Humphreys and Ruseski, 2007), and county government PRS expenditures are linked with better self-rated health and lower mortality (Mueller et al., 2019a; Mueller et al., 2019b). No prior research, however, has examined benefits of PRS spending on birth outcomes. Such a link is plausible given that residential greenness and proximity to parks are inversely associated with LBW incidence (Banay et al., 2017; Grazuleviciene et al., 2015; Seabrook et al., 2019), partly via greater maternal physical activity and reduced maternal depression (McEachan et al., 2015; South et al., 2018). Access to green spaces and recreational programs can foster positive developmental and educational outcomes among youth (Eccles et al., 2003; Pfeifer and Cornelissen, 2010; Campbell et al., 2018), promote cognitive functioning in childhood and adulthood (Taylor et al., 2002; Wells, 2000; Berman et al., 2008), assist with stress coping (Kondo et al., 2018), and facilitate increased physical activity (Cawley et al., 2007; Humphreys and Ruseski, 2007). Such diverse benefits suggest that increasing PRS could indirectly lead to better population birth outcomes.

Expanding access to public parks and recreational opportunities may influence black-white differences in birth outcomes. Black Americans tend to have low access to safe, high-quality parks relative to whites (Dahmann et al., 2010; Dai, 2011; Taylor et al., 2007), even where park proximity differences do not exist (Wen et al., 2013). Moreover, given that black Americans are more likely to experience household and neighborhood poverty, lack of access to private recreational opportunities is a potential barrier to regular physical activity and positive youth development (*The Aspen Institute*, 2018). Expanding public PRS could therefore address unmet needs and disproportionately improve the health of black Americans. Evidence indicates that self-reported access to parks and recreational facilities may be more predictive of obesity risk for black relative to white children (Alexander et al., 2013). However, black Americans tend to use parks less often than whites, potentially due to lower spatial access, social norms, racial bias in park design, or feelings of exclusion (Byrne and Wolch, 2009), such that exclusionary PRS expansions could exacerbate existing inequities.

Another health-relevant public service is housing and community development (HCD) programs—e.g., renewing urban centers, increasing affordable housing stock, and offering housing vouchers (Howell, 2016). Because affordable housing in safe neighborhoods is a key health resource (Evans et al., 2003; Krieger and Higgins, 2002), and neighborhood economic disadvantage, housing instability, and homelessness increase LBW risk (Carrion et al., 2015; Metcalfe et al., 2011; Richards et al., 2011), effective HCD initiatives may lead to improved population birth outcomes. Plausible mechanisms linking housing quality to maternal health, and, in turn to LBW, include exposure to physical hazards (e.g., smoke, mold) and maternal chronic stress (Shaw, 2004; Taylor et al., 1997).

Moreover, HCD programs could differentially influence LBW incidence for black and white infants. Black adults are more likely to reside in economically disadvantaged urban centers relative to whites (Massey and Denton, 1993; Williams and Collins, 2001) and to be recipients of public housing (Goetz, 2011). Racial residential patterns are also starkly segregated in many US counties, leading to under-resourced black communities with higher risks for LBW (Mehra et al., 2017). Thus, stemming from black Americans' greater enrollment in assistance programs and investment needs in predominantly black communities, HCD programs represent one policy lever that could produce greater equity in outcomes between black and white infants. However, HCD programs that are disruptive to established communities could have unintended, adverse effects (e.g., loss of social networks; required but undesired residential moves) (Goetz, 2011).

The current study examines whether changes in local government expenditures for [1] PRS and [2] HCD influence county LBW outcomes—specifically, the LBW incidence rate and the gap in LBW between non-Hispanic blacks and whites. These analyses add to extant literature by offering the first test of the benefits to birth outcomes of local government expenditures on PRS and HCD. Moreover, the explicit focus on black-white differences in LBW introduces a novel policy-relevant predictor to a longstanding research topic. Identifying health-promoting local government practices could generate innovation around budgeting and policy initiatives aimed at improving birth outcomes and reducing racial disparities.

## 2 Methods

### 2.1 Data

**Fig. 1:**
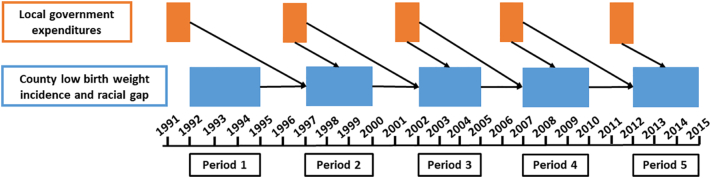
Timeline of assessments for government expenditures and birth outcomes, with arrows indicating modeled associations. *Notes.* Expenditures occurred in the fiscal year preceding the survey year. Fiscal years are specific to each local government, concluding between July 1 of the previous year through June 30 of the survey year.

Data were derived from multiple sources. National birth records with county identifiers were obtained from the National Center for Health Statistics, spanning the period of 1992–2014. Government expenditures were available from the U.S. Census Bureau's Census of Governments, administered every five years to all counties, municipalities, townships, and special districts (~approximately 87,000 local governments) (Pierson et al., 2015). Survey years 1992, 1997, 2002, 2007, and 2012 were included, with LBW outcomes from ensuing periods being selected to improve the temporal ordering of variables (see Fig. 1 for timeline of measurement occasions). County median household income and population estimates were available through Census Bureau programs (Small Area Income and Poverty Estimates and Housing Unit and Population Estimates, respectively).

The sample was refined to singleton births to non-Hispanic white or non-Hispanic black mothers. Eligibility criteria were based on differences in etiology of LBW for singleton and multiple gestation births, and due to our focus on black-white disparities. Of 60.68 million total births in the U.S. during the five assessment periods, 41.02 were singletons to non-Hispanic black or white mothers. Three-year pooled estimates of county-level LBW outcomes were computed and represent: 1992–1994; 1997–1999; 2002–2004; 2007–2009; and 2012–2014. The following inclusion criteria were then applied to counties: at least ten cases of LBW for each racial group (i.e., black and white) per period to improve reliability; two consecutive periods with concurrent and lagged data; and available data for county variables. The cutpoint of ten LBW cases is similar to prior research (Crosse et al., 1997; Tu et al., 2012). Inclusion criteria resulted in an analytic sample of 956 counties (30.4% of 3142 total counties), with an average of 3.8 of 4 observations and a total of 3619 county-by-period observations (the first of five periods was only included as a lag). These observations represent approximately three-quarters of U.S. singleton births to black or white mothers of non-Hispanic origin (i.e., 24.08 of 32.85 million total births in the final four periods). Sample counties are depicted in Figs. S1 and S2 of Supplemental Material, and sample biases from exclusion criteria are described in the Results.

### 2.2 Measures

#### 2.2.1 Low birth weight

Birth weight is an appropriate focus for the present research for methodological and clinical reasons. LBW (i.e., <2500 g) is common relative to infant mortality and reliably measured, important for estimating small area incidence rates. Also, LBW is a major contributor to neonatal mortality and life course outcomes, such as lower educational attainment and higher cardiovascular disease, especially for births <1500 g (Petrou et al., 2001; Boardman et al., 2002; Almond and Currie, 2011; Belbasis et al., 2016). Finally, black-white differences in LBW are substantial and account for the predominant share of the infant mortality gap (MacDorman and Mathews, 2011).

LBW was coded from individual records as <2500 g (Northam and Knapp, 2006). Maternal race and Hispanic ethnicity were self-reported (Ingram et al., 2003), and classifications of non-Hispanic white and non-Hispanic black were used to compute race-specific LBW incidence. Records were aggregated to county of maternal residence and pooled over three-year-periods as LBW incidence per 100 singleton live births among black and white infants, and absolute difference in LBW incidence between black and white infants. Parallel very LBW outcomes were coded using the criterion of <1500 g and considered in supplemental models.

#### 2.2.2 Local government expenditures

Two expenditure categories were of primary interest: (1) PRS (e.g., park maintenance, provision of recreational and cultural-scientific facilities); and (2) HCD (e.g., rent subsidies, promotion of home ownership, urban renewal) (Pierson et al., 2015). Three expenditure categories were modeled as covariates to reduce potential confounding: total operational costs; health (e.g., public health administration, community health care, health education, mental health services, regulation of air/water); and hospitals (e.g., government's own hospitals and spending for provision of care in public or private hospitals, excluding payments for medical services via welfare or medical assistance programs). Detailed description of expenditure categories is available at https://www2.census.gov/govs/pubs/classification/2006_classification_manual.pdf.

Expenditures were defined as operational costs, including direct employee compensations and costs for supplies, materials, and contractual services. Operational costs financed through grants or transfers were included while intergovernmental transfers to other local governments were excluded to avoid double counting. Operational costs are stable year-to-year relative to capital outlay, such that variation likely reflects distinct shifts in priorities and service provision (Kaczynski and Crompton, 2006; Jordan, 2003). Expenditure levels are not a direct measure of service provision, however, and may reflect only new overhead costs. County expenditures were summed for county and sub-county general purpose and special purpose governments (i.e., municipalities, townships, special districts). Modeling all local government expenditures more accurately measures local service provision, particularly in places where county governments serve a relatively limited role (Mueller et al., 2019b; Schneider and Park, 1989). Values were adjusted for population and inflation by converting expenditures to per capita 2012 dollars.

#### 2.2.3 County sociodemographic covariates

County median household income, percent of total residents who are black (i.e., black density), and population change were included as time-varying covariates. Median household income estimates come from Small Area Income and Poverty Estimates, based on administrative tax records, government transfers, and Census Bureau data (Bell et al., 2007). Where possible, inflation adjusted three-year averages were computed (1993, 1997–1999, 2002–2004, 2007–2009, and 2012–2014). Population estimates and black density correspond to the midpoint of birth outcome periods. Percentage change in total population was coded as: Δ population from prior period/population in prior period.

### 2.3 Analyses

First, descriptive statistics, histograms, and Pearson correlation coefficients were examined for raw and county mean-centered variables. Outlier data points were winsorized at 4 SD units to reduce violations of linear model assumptions.

For study hypotheses, fixed effects models were employed wherein time-invariant between-county differences and period effects were removed. This approach reduces omitted variable bias as stable between county differences are controlled (Allison, 2009). To model change, the lagged dependent variable was included in all models. Including the lagged dependent variable in a fixed time series with small number of periods results in the Nickell bias, arising from a correlation between the lagged dependent variable and the unit-period specific error term (Nickell, 1981). To correct for this downward bias of coefficients, bootstrap-based bias-corrected fixed effects models were fit using the XTBCFE command in STATA v. 14.2 (De Vos et al., 2015; Everaert and Pozzi, 2007). Two hundred bootstrap samples were used for bias correction, and 100 bootstrap samples to estimate standard errors. A randomized temporal heteroscedasticity resampling scheme was selected, which resamples over time and within cross-sections and is appropriate for short time series and cross-sectional dependence (De Vos et al., 2015).

Model progression was similar for both outcomes: LBW incidence and black-white gap in LBW. Concurrent local government expenditures (i.e., HCD, PRS, total, health, and hospitals) were entered as predictors of LBW incidence, alongside the lagged dependent variable and period dummies (Model 1). Next, lagged local government expenditures for each of the five categories were added (Model 2). Model 3 was further adjusted for time-varying county sociodemographic covariates, and is shown by the following equation:

$$Y_{\mathrm{ti}}=a_{i}+{\mathrm{\gamma Y}}_{\left(t-1\right)i}+b_{1}{\mathrm{HCD}}_{\mathrm{ti}}+b_{2}{\mathrm{HCD}}_{\left(t-1\right)i}+b_{3}{\mathrm{PRS}}_{\mathrm{ti}}+b_{4}{\mathrm{PRS}}_{\left(t-1\right)i}+\beta_{5}x_{\mathrm{ti}}+\beta_{6}\mathrm{period}+e_{\mathrm{ti}},$$

where *Y*_*ti*_ refers to LBW incidence at period t in county i; *a*_*i*_ is the time-invariant county effect; *Y*_(*t*−1)*i*_ is LBW incidence lagged by one period; *γ* is the autoregressive coefficient for lagged LBW incidence; *b*_1_ through *b*_4_ refer to coefficients representing the association between housing and community development (HCD) and parks and recreation (PRS) expenditures and LBW incidence, *β*_5_*x*_*ti*_ is a vector of concurrent and lagged time-varying covariates; *β*_6_*period* is a vector for period dummy variables; and *e*_*ti*_ is the error for period t in county i. The second series of models (Models 4, 5, and 6) followed a parallel progression with black-white absolute gap in LBW as the outcome.

Alternative model specifications were considered (e.g., different model progression, two period time lags, more restrictive inclusion criteria), and models were fit using very LBW outcomes. Further description and presentation of results are included in Supplemental Material.

## 3 Results

County descriptors by period are shown in Table 1. Relative to excluded counties, sample counties (*N* = 956) were more populous, and had higher black density, median household income, and LBW incidence (using county mean values; *p* values < .001). Differences in the magnitude of the racial gap in LBW were not considered due to unreliable estimates among excluded counties. Across the study period, mean local government total operational costs were $3235 per capita in 2012 dollars, and only relatively minor shares of expenditures were for HCD (1.9%) and PRS (1.5%). Average HCD expenditures increased from $43 to $74 per capita between 1992 and 2012, and PRS expenditures increased from $38 to $53. Although descriptive statistics indicated skewed distributions for several of the expenditure variables, within county distributions of variables were approximately normally distributed. Within county fluctuations in total operational costs were weakly-to-moderately correlated with HCD, PRS, health, and hospital expenditures (*r* coefficients ranged from 0.23 to 0.55), whereas fluctuations between expenditure types were at most weakly correlated (*r*s < 0.17).

**Table 1:** Descriptive statistics for 956 United States counties in study periods.

	Period 1	Period 2	Period 3	Period 4	Period 5	Within County
Variables	M ± SD	M ± SD	M ± SD	M ± SD	M ± SD	±SD
Local government expenditures ($100 s per capita, 2012 dollars)						
Total operational	27.04 ± 9.45	28.91 ± 9.55	32.99 ± 11.24	36.72 ± 13.12	36.04 ± 12.44	±5.76
Housing and community	0.43 ± 0.50	0.51 ± 0.53	0.63 ± 0.63	0.70 ± 0.67	0.74 ± 0.70	±0.32
Parks and recreation	0.38 ± 0.37	0.41 ± 0.38	0.50 ± 0.43	0.55 ± 0.49	0.53 ± 0.44	±0.16
Health	0.63 ± 0.69	0.75 ± 0.97	0.86 ± 1.08	0.92 ± 1.10	0.91 ± 1.10	±0.46
Hospital	2.34 ± 3.98	2.26 ± 4.37	2.36 ± 5.03	2.54 ± 5.49	2.73 ± 5.81	±2.27
Total population (in 10,000 s)	20.25 ± 45.68	21.49 ± 47.74	22.66 ± 50.07	23.80 ± 51.32	24.90 ± 53.68	±4.71
Black density (%)	19.81 ± 15.17	20.20 ± 15.41	20.00 ± 15.42	20.19 ± 15.46	20.23 ± 15.41	±1.65
Mdn income ($1000s, in 2012)^a^	47.20 ± 13.00	50.62 ± 13.48	48.97 ± 13.86	49.62 ± 14.48	46.69 ± 13.78	±2.74
Low birth weight (per 100)	6.85 ± 1.80	7.05 ± 1.77	7.45 ± 1.97	7.70 ± 2.10	7.41 ± 2.00	±0.83
Racial gap in LBW (per 100)	6.08 ± 2.47	5.90 ± 2.17	6.18 ± 2.21	6.02 ± 2.21	5.82 ± 2.43	±1.90

*Notes.* LBW = low birth weight.

a The mean value (across counties) is reported for the median (Mdn) income within counties.

### 3.1 County low birth weight incidence

**Fig. 2:**
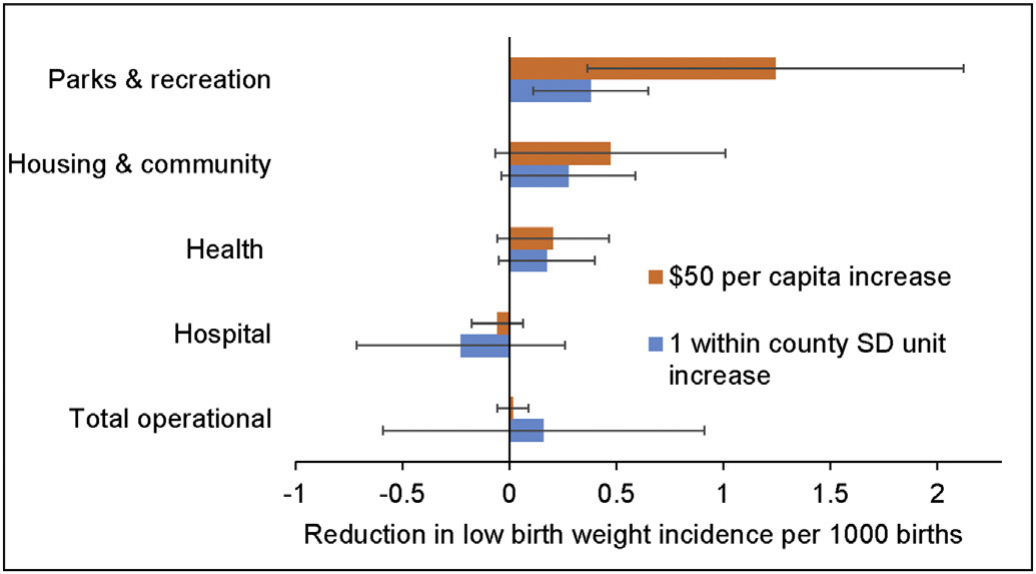
Estimated magnitude of association between changes in local government expenditures and county low birth weight incidence. *Notes.* Estimated association derived from Model 3. To depict statistical significance, 95% confidence intervals are shown and bars crossing 0 indicate non-significance.

Results are shown for regression models in Table 2. Adjusting for prior LBW incidence, county and period effects, and concurrent total operational, health, and hospital expenditures, Model 1 results indicate that higher PRS expenditures relative to the county mean were associated with a decrease in LBW incidence. This estimate was not substantively altered when adjusting for lagged expenditures (Model 2; variables were already county mean centered). The estimate is equivalent to higher PRS expenditures of $50 per capita being linked to 1.25 fewer LBW cases per 1000 (*p* = .012), or 0.18 within county SD units in LBW. Fluctuations in HCD expenditures were not significantly associated with LBW incidence in either model. The estimate was in the expected direction but the confidence intervals included 0. When adjusting for county time-varying median household income, black density, and population change (Model 3), the association between PRS expenditures and LBW incidence was of comparable magnitude to earlier estimates and significant. The estimated effect of a $50 per capita increase (and a within county 1 SD unit increase) on LBW incidence is shown for each of the government expenditure types in Fig. 2.

**Table 2:** Estimates from bias corrected fixed effects models indicating the influence of local government expenditures on changes in county low birth weight incidence per 100 births (*N* = 956 United States counties, 3619 observations).

	Model 1		Model 2		Model 3	
	Est.	[95% CI]	Est.	[95% CI]	Est.	[95% CI]
Low birth weight_(*t*__−__1)_	**0.33**	**[0.25, 0.41]**	**0.33**	**[0.25, 0.41]**	**0.27**	**[0.19, 0.35]**
Local government expenditures (Δ $100 per capita)						
Parks and recreation	**−**‐****0.25****	**[−0.44, −0.06]**	**−0.26**	**[−0.44, −0.08]**	****−**0.25**	**[−0.42, −0.07]**
Housing and community	−0.08	[−0.20, 0.03]	−0.09	[−0.20, 0.02]	−0.09	[−0.20, 0.01]
Health	−0.05	[−0.10, 0.01]	−0.05	[−0.11, 0.01]	−0.04	[−0.09, 0.01]
Hospitals	0.01	[−0.01, 0.03]	0.01	[−0.01, 0.03]	0.01	[−0.01, 0.04]
Total operational	0.00	[−0.02, 0.01]	0.00	[−0.02, 0.01]	0.00	[−0.02, 0.01]
Parks and recreation_(*t*__−__1)_			−0.02	[−0.21, 0.18]	−0.03	[−0.20, 0.14]
Housing and community_(*t*__−__1)_			−0.01	[−0.12, 0.10]	−0.01	[−0.12, 0.09]
Health_(*t*__−__1)_			−0.04	[−0.10, 0.03]	−0.03	[−0.09, 0.03]
Hospitals_(*t*__−__1)_			−0.01	[−0.04, 0.01]	−0.01	[−0.03, 0.01]
Total operational_(*t*__−__1)_			0.01	[−0.01, 0.02]	0.01	[−0.01, 0.02]
Demographic and economic covariates						
Median household income ($10,000)					**−0.14**	**[−0.26, −0.03]**
Black density (10%)					**0.70**	**[0.46, 0.94]**
Population change (10%)					**−0.16**	**[−0.26, −0.05]**

*Note.* Estimates in bold are significant at *p* < .05. Period and county fixed effects are included in all models.

### 3.2 Racial gap in low birth weight incidence

Model results for the county black-white gap in LBW are shown in Table S1 of Supplemental Material. Neither HCD nor PRS expenditures was associated with the county racial gap in LBW, whether expenditures were measured lagged, concurrently, or as change between periods. Of the other expenditures, lagged health expenditures were associated with a shrinking racial gap in LBW incidence over an approximately five-year period (*p* < .001), with a $50 increase per capita being associated with the racial gap in LBW closing by 1.46 cases per 1000; this finding is discussed in the Supplemental Material.

## 4 Discussion

We find an increase in local government PRS expenditures over a five-year-period to be associated with a decrease in county LBW incidence. Specifically, an additional annual investment of $50 per capita on PRS reduced LBW incidence by 1.25 cases per 1000 live births. Although a small effect, such a reduction amounts to approximately 1/3 of the Healthy People 2020 goal for reducing national LBW incidence (i.e., 4 fewer cases per 1000), and is therefore of considerable value at the population level. Our study findings are broadly consistent with research on the salubrious effects of residential greenness and green spaces on maternal health and pregnancy outcomes (Banay et al., 2017). The majority of this research has investigated residential greenness (i.e., density of vegetation), however, with relatively little attention to the association between parks and birth outcomes (Grazuleviciene et al., 2015); our findings demonstrate the value of such research. Given the substantial public resources allocated to PRS—albeit arguably insufficient (Godbey et al., 2010)—it is important that the wide-ranging benefits are understood.

One potential explanation for the benefits of PRS spending on LBW is via improvements in maternal health in preconception and prenatal periods (e.g., by increasing physical activity and social interaction). PRS expenditures have been shown to increase exercise and time spent in outdoor recreation (Cawley et al., 2007; Humphreys and Ruseski, 2007), and to improve self-rated health (Mueller et al., 2019a), with the potential to indirectly benefit birth outcomes (Mueller et al., 2019b; Banay et al., 2017; Leiferman and Evenson, 2003; Scientific Report, n.d.). Another potential mechanism for the effects of increases in parks and recreation services on LBW risk is through influencing fertility timing and patterns. Prior research has shown that involvement in recreational activities can encourage positive youth development (Eccles et al., 2003; Fraser-Thomas et al., 2005), manifesting in fewer risky behaviors, the formation of social skills, and higher educational attainment (Pfeifer and Cornelissen, 2010; Cohen et al., 2007). Such accrued developmental benefits have been associated with fewer pregnancy risks (Kramer et al., 2000; Cohen et al., 2007; Gilman et al., 2008). Specific pathways through which PRS expenditures influence LBW risk and other adverse birth outcomes is an important topic for future research.

We did not find a significant association between HCD expenditures and reduced county LBW incidence rate. Although voluminous research exists documenting the health benefits of stable and safe housing (Carrion et al., 2015; Burgard et al., 2012; Srinivasan et al., 2003), including from increasing HCD expenditures (McCullough and Leider, 2016), the benefits of HCD programs on maternal and infant health are not well understood. Research is especially needed that compares the effects of specific local government HCD programs on the risk for adverse birth outcomes (Slopen et al., 2018). Accordingly, our analytic strategy was limited by the use of a broad category of HCD expenditures. Based on prior findings, providing housing vouchers to assist with relocation from public housing, investing in disadvantaged communities, and equitable zoning policies are a few strategies that could be implemented by local governments with supportive evidence for health benefits (Maantay, 2001; Sanbonmatsu et al., 2011; Wolch et al., 2014), although gains to maternal and infant health are not understood.

Study strengths include a large sample of counties with multiple waves of data to model within county effects; the use of a large sample of births; and a novel test of the link between local government expenditures and LBW, with an additional focus on county black-white disparities. Nonetheless, study limitations remain. Although the sample of counties included nearly three-quarters of singletons to non-Hispanic white or black mothers, the findings should not be generalized to all U.S. counties. Sample counties tended to be in urban areas located in the Southeastern U.S., and include substantial black populations. Future research should investigate how the association between PRS expenditures and LBW incidence varies by region and urban-rural status.

Available data did not allow for modeling of the spatial distribution of new PRS or HCD expenditures. The extent to which services are inequitably distributed would limit the potential for new services to reduce health disparities. For example, one study found that Los Angeles neighborhoods with predominant ethnic minority populations have fewer parks despite denser populations and that new parks exacerbated existing inequities in park access (Wolch et al., 2005). Similarly, data did not allow for examination of different program types, limiting the conclusions we could draw. For example, the provision of housing assistance may have been through public housing opportunities—often situated in high poverty, spatially disconnected communities—or through housing vouchers that allow for movement into better-resourced mixed income communities (Sanbonmatsu et al., 2011). Examples abound of marginalized populations being displaced as a result of urban redevelopment or of investments benefiting suburban communities at the expense of densely populated urban centers where a disproportionate share of people of color reside (Goetz, 2011; Thomas, 2013). Thus, although present analyses offer an important examination of government expenditures, future research needs to identify specific local governments practices that are successful in promoting health via PRS or HCD, especially with attention to access for disadvantaged communities.

Another limitation is that our modeling strategy did not allow for investigation of between county differences in hypothesized associations. Local government features and the socioeconomic profile of residents influence government spending patterns and thereby the impact on population health. The focus on within county variance over time was preferred as area characteristics are highly collinear, introducing the potential for multiple confounders and limiting the reliability of estimates (Morgenstern, 1995). As such, controlling for stable county characteristics reduced the possibility of omitted variable bias. Although we adjusted for select time-varying confounders in the link between local government expenditures and LBW incidence, we cannot rule out the possibility that important confounders were unintentionally excluded (e.g., changes in public green space or residential greenness that may accompany greater investment in PRS).

## 5 Conclusion

The health implications of local government practices and policies are infrequently the focus of scientific inquiry. The role of local governments as service providers, however, has been expanding and the expectations for improvements to human health should follow, particularly among disadvantaged population groups. The current study finds evidence that increases in local government expenditures on PRS are associated with reductions in LBW incidence, independent of changes in total spending and other health-relevant services. If confirmed by future research, policies that focus on increasing PRS expenditures could be used to improve birth outcomes. Given that reductions in LBW incidence would decrease many associated societal costs and have national externalities (Petrou et al., 2001; Almond et al., 2005), there is a need for state and federal grants to support communities that lack sufficient PRS. Factoring in the indirect population health gains of PRS alongside primary intended benefits (e.g., increasing exercise) presents a more complete accounting of PRS benefits and may increase public appetite for such services.

## Declaration of competing interest

None.

## Appendix A Supplementary material

Supplementary material Image 1
