# Supplementary material for: Associations between local government expenditures and low birth weight incidence: Evidence from national birth records
Source: Prev Med Rep. 2019 Aug 30;16:100985. doi: 10.1016/j.pmedr.2019.100985 (PMC6734050; doi:10.1016/j.pmedr.2019.100985)
Supplement: Supplementary file 1 — Supplementary material [file mmc1.docx]

**SUPPLEMENTAL MATERIAL**

**Methods**

Several alternative models specifications were considered. First, HCD and PRS expenditures were examined in separate models, adjusting for county and period effects, total expenditures, and county sociodemographic covariates. Second, alternative lag specifications were modeled with reference to both LBW outcomes. Specifically, dependent variables and local government expenditures were lagged for two periods (i.e., *t*, *t*-1, *t*-2) to examine the long-run impact of PRS and HCD. The additional lag resulted in a loss of one period and reduced the sample of counties to 899 with 2606 total observations. Third, models were fit using more restrictive inclusion criteria, such that all counties had at least 20 cases of LBW for each racial group per period (*n* = 700 counties and 2611 county-by-period observations). This third approach was utilized to improve the reliability of estimates of the black-white LBW gap.

Very low birth weight incidence (VLBW; i.e., number of births of less than 1500 g. per 1000 live births) and the black-white gap in VLBW incidence were also considered as outcome variables in supplemental models. Examination of VLBW incidence leads to a substantial reduction in the analytic sample of counties (*n* = 387) because of the inclusion criteria that counties have at least ten cases of VLBW for both black and white infants. The advantage of examining VLBW, however, is that the immediate clinical significance and long-term sequelae of VLBW are much greater relative to cases of moderate low birth weight (1500 – 2499 g.) (1,2). Moreover, the relative racial disparity in VLBW is approximately three-fold and is the largest contributor to racial differences in infant mortality (3). We present results for both LBW and VLBW incidence to capitalize on the advantages of each outcome.

**Results**

**Alternative Model Specifications**

In alternative models where PRS and HCD were examined separately—adjusting for time and county effects, total operational costs (*t*, *t*-1), and county income and demographic covariates—results were similar to the primary models but indicated a significant effect of HCD expenditures. Specifically, an increase of $50 in HCD expenditures led to .60 fewer LBW cases per 1000 (95% CI: -1.15, -0.06; *p* = .030). An increase of $50 in PRS expenditures reduced LBW incidence by 1.15 cases per 1000 (95% CI: -2.02, -0.27; *p* = .010). When PRS and HCD were added separately as predictors of the black-white gap in LBW, neither variable was a significant predictor.

When modeling the long-term results of PRS expenditures, results indicated that neither PRS nor HCD expenditures from two periods earlier were associated with the approximate ten-year change in LBW incidence (*p* = .29 and *p* = .70, respectively). In this model, however, concurrent PRS expenditures were associated with reduced LBW (*p* = .007) and the long-run impact of PRS expenditures (computed as the sum of *t*, *t* - 1, *t* – 2) was relatively large and significant; the estimated ten-year impact of a $50 increase per capita in PRS expenditures is 4.41 fewer LBW cases per 1000 births (Wald χ^2^ = 3.01 [3, 1685], *p* = .029). Considering multiple lags likely introduces bias into estimates due to overspecification, particularly with a short time series, such that these results should be interpreted cautiously but merit further research. The long-run impact of HCD expenditures was not significant (Wald χ^2^ = 0.42 [3, 1685], *p* = .74). With relation to the long-term results of PRS and HCD expenditures on the racial gap in LBW incidence, none of the PRS or HCD expenditure variables (*t*, *t* - 1, *t* – 2) were associated with changes in the racial gap in LBW incidence.

When more restrictive inclusion criteria were implemented requiring 20 cases of LBW for each racial group (n=700 counties, 2611 county-by-period observations), findings were similar in direction but estimates were smaller in magnitude and nonsignificant. In particular, adjusting for covariates listed in Model 1, a $50 increase in PRS had an estimated impact of .79 fewer LBW cases per 1000 live births (95% CI: -1.98, 0.41, *p* = .20). The equivalent estimate for HCD was also nonsignificant (*p* = .52). Among this sample, neither HCD nor PRS expenditures was associated with the black-white gap in LBW.

**Very Low Birth Weight Outcomes**

The analytic sample was reduced to 387 counties with the total number of observations being 1427. Regression model results for VLBW incidence are shown in Table S2. In model 3, adjusting for other expenditures_(_*_t_*_,_ *_t_* _-_ _1)_ and county demographic and economic covariates, concurrent PRS expenditures were not significantly associated with VLBW incidence but the estimated association was in the expected direction (Est. = -.31, 95% CI: -.74, .13). A $50 increase per capita in PRS expenditures was estimated to reduce the incidence per 1000 live births by .15 cases, equivalent to .89 SD units of within county variance in VLBW.

Model results for county black-white differences in VLBW incidence are shown in Table S3. Findings were generally similar to models considering the racial gap in LBW. Specifically, neither lagged nor concurrent HCD or PRS expenditures was associated with the racial gap in VLBW incidence, yet lagged health expenditures_(_*_t_* _– 1)_ was associated with a declining racial gap in VLBW incidence (Est. = -.65, 95% CI: -1.25, -.05). Higher per capita spending of $50 on health services had a lagged impact on the black-white VLBW gap, such that the disparity was reduced by .33 cases per 1000 live births.

**Discussion**

The impact of local government health expenditures was not a primary focus of the current study, yet the reported findings on this topic warrant discussion. Higher health expenditures in the prior period were associated with a shrinking racial gap in LBW and VLBW incidence over an approximately five-year period. Prior research on the link between local health department spending and black-white disparities in adverse birth outcomes has been inconclusive. Specifically, prior findings suggest that health expenditures may reduce infant mortality to a greater degree among black relative to white infants (4), and that counties providing family planning, prenatal care, and nutritional assistance services marginally reduced their black-white infant mortality gap over time relative to counties without these services (5); however, neither of these prior findings was statistically significant. In contrast, a more recent study that included data on specific public health programs found that targeted infant health expenditures reduced infant mortality to a larger degree among blacks relative to whites (6).

Health expenditures in the present study were not significantly associated with reduced county incidence of LBW, yet findings were in the expected direction with a *p*-value of approximately .10. The estimated effect of health expenditures is likely biased downwards, however, due to the broad category of health included rather than maternal health and family planning programs that would have a larger impact on birth outcomes (7)—although total health department spending has been linked with lower rates of LBW and infant mortality (4,7). Our findings are broadly consistent with this research and extend these findings to a substantially larger sample of counties and across multiple periods. Additional research is needed to identify effective programs for local health departments to improve birth outcomes while reducing associated racial disparities, yet our findings demonstrate the importance of public health expenditures for improving population birth outcomes.


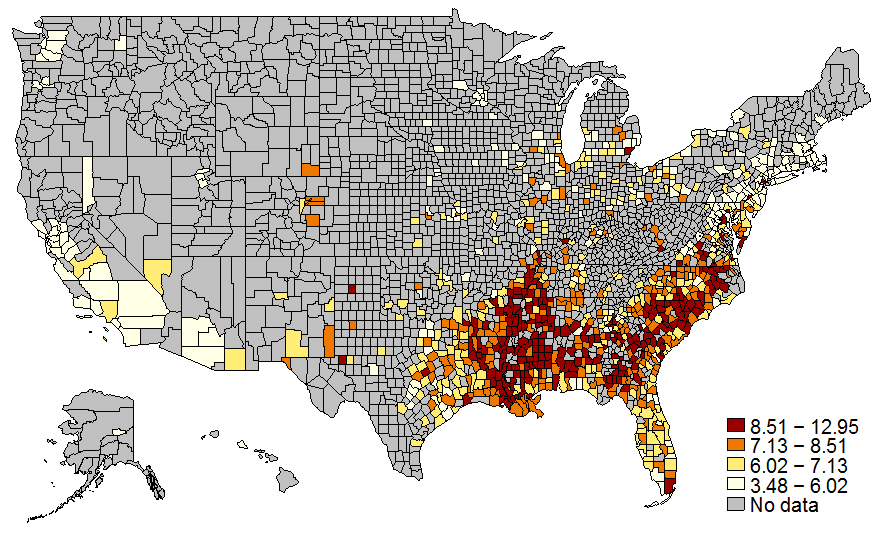


Figure S1. County low birth weight incidence per 100 births among black and white infants for counties included in primary analyses (N=956 counties), averaged across five measurement occasions between 1992 to 2014.


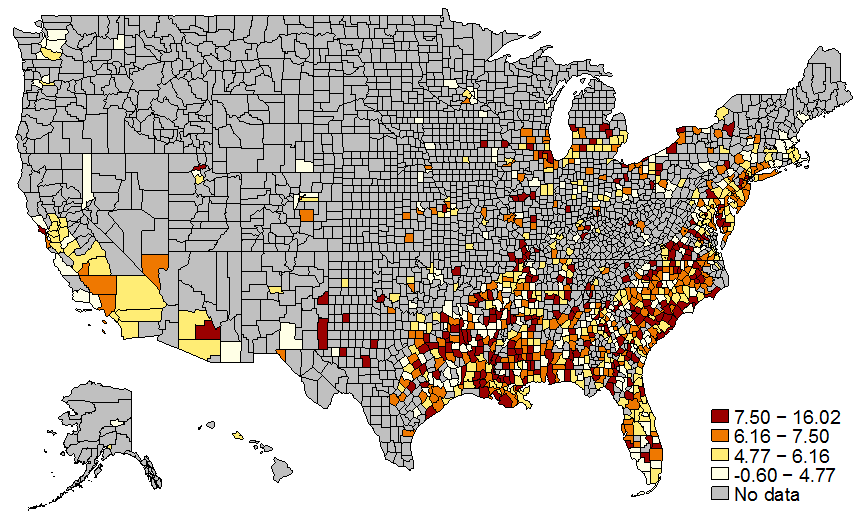


Figure S2. Racial gap in county low birth weight incidence per 100 births between black and white infants for counties included in primary analyses (*N*=956 counties), averaged across five measurement occasions between 1992 to 2014.

Table S1. Estimates from bias corrected fixed effects models indicating the influence of local government expenditures on changes in county black-white differences in low birth weight (LBW) incidence per 100 births (*N* = 956 counties for a total of 3619 observations).

|  | Model 4 | | Model 5 | | Model 6 | |
| --- | --- | --- | --- | --- | --- | --- |
|  | Est. | [95% CI] | Est. | [95% CI] | Est. | [95% CI] |
| Racial gap in LBW_(_*_t_*_-1)_ | **.15** | **[.09, .21]** | **.15** | **[.08, .21]** | **.14** | **[.08, .20]** |
| Local government expenditures (Δ $100 per capita) | | |  |  |  |  |
| Parks and recreation | -.23 | [-.74, .28] | -.23 | [-.74, .28] | -.21 | [-.76, .34] |
| Housing and community | -.06 | [.32, .21] | -.03 | [-.29, .22] | -.03 | [-.29, .22] |
| Health | .04 | [-.11, .18] | .02 | [-.12, .16] | .02 | [-.13, .16] |
| Hospitals | .03 | [-.02, .08] | .03 | [-.02, .03] | .03 | [-.02, .08] |
| Total operational | -.01 | [-.04, .02] | -.01 | [-.03, .02] | -.01 | [-.03, .02] |
| Parks and recreation_(_*_t_*_-1)_ |  |  | -.05 | [-.55, .44] | -.02 | [-.53, .49] |
| Housing and community_(_*_t_*_-1)_ |  |  | .04 | [-.25, .33] | .05 | [-.27, .36] |
| Health_(_*_t_*_-1)_ |  |  | **-.29** | **[-.44, -.14]** | **-.30** | **[-.47, -.12]** |
| Hospitals_(_*_t_*_-1)_ |  |  | .01 | [-03, .05] | .00 | [-.04, .04] |
| Total operational_(_*_t_*_-1)_ |  |  | -.01 | [-.04, .01] | -.01 | [-.03, .02] |
| Demographic and economic covariates | |  |  |  |  |  |
| Median household income ($10,000) | |  |  |  | -.27 | [-.57, .03] |
| Black density (10%) |  |  |  |  | -.36 | [-.79, .06] |
| Population change (10%) |  |  |  |  | **-.25** | **[-.50, -.01]** |

*Note.* Estimates in bold are significant at *p* <.05. Period and county fixed effects are included in all models.

Table S2. Estimates from bias corrected fixed effects models indicating the influence of local government expenditures on changes in county incidence of very low birth weight (VLBW) per 1000 live births *(n* = 387 counties; 1427 observations).

|  | Model 1 | | Model 2 | | Model 3 | |
| --- | --- | --- | --- | --- | --- | --- |
|  | Est. | [95% CI] | Est. | [95% CI] | Est. | [95% CI] |
| VLBW_(_*_t_*_-1)_ | **.44** | **[.27, .60]** | **.44** | **[.27, .61]** | **.22** | **[.09, .36]** |
| Local government expenditures ($100 per capita) | | |  |  |  |  |
| Parks and recreation | -.32 | [-.82, .18] | -.29 | [-.79, .20] | -.31 | [-.74, .13] |
| Housing and community | .13 | [-.23, .48] | .11 | [-.30, .53] | .10 | [-.26, .46] |
| Health | -.02 | [-.21, .16] | -.02 | [-.21, .17] | .02 | [-.16, .20] |
| Hospitals | .02 | [-.04, .09] | .02 | [-.06, .10] | .03 | [-.03, .09] |
| Total operational | -.02 | [-.05, .01] | -.02 | [-.05, .02] | -.02 | [-.05, .01] |
| Parks and recreation_(_*_t_*_-1)_ |  |  | .20 | [-.34, .74] | .22 | [-.32, .76] |
| Housing and community_(_*_t_*_-1)_ |  |  | .00 | [-.34, .34] | .06 | [-.30, .43] |
| Health_(_*_t_*_-1)_ |  |  | -.04 | [-.27, .18] | -.03 | [-.23, .16] |
| Hospitals_(_*_t_*_-1)_ |  |  | .01 | [-.05, .08] | .01 | [-.04, .07] |
| Total operational_(_*_t_*_-1)_ |  |  | .00 | [-.03, .03] | .01 | [-.02, .03] |
| Demographic and economic covariates | |  |  |  |  |  |
| Median household income ($10,000) | |  |  |  | -.26 | [-.59, .07] |
| Percent black (10%) |  |  |  |  | **2.76** | **[1.92, 3.61]** |
| Population change (10%) |  |  |  |  | **-.54** | **[-.89, -.19]** |

*Note.* Estimates in bold are significant at *p* <.05. Period and county fixed effects are included in all models.

Table S3. Estimates from bias corrected fixed effects models indicating the influence of local government expenditures on changes in county black-white differences in incidence of very low birth weight per 1000 live births *(n* = 387 counties; 1427 observations).

|  | Model 4 | | Model 5 | | Model 6 | |
| --- | --- | --- | --- | --- | --- | --- |
|  | Est. | [95% CI] | Est. | [95% CI] | Est. | [95% CI] |
| Racial gap in VLBW_(_*_t_*_-1)_ | .05 | [-.05, .14] | .05 | [-.05, .14] | .05 | [-.06, .15] |
| Local government expenditures ($100 per capita) | | |  |  |  |  |
| Parks and recreation | .14 | [-.96, 1.24] | .18 | [-.89, 1.26] | .25 | [-.85, 1.36] |
| Housing and community | .23 | [-.60, 1.06] | .15 | [-.71, 1.01] | .18 | [-.77, 1.12] |
| Health | .05 | [-.42, .52] | .08 | [-.38, .55] | .06 | [-.41, .53] |
| Hospitals | **.11** | **[.01, .21]** | .10 | [-.01, .21] | .09 | [-.05, .22] |
| Total operational | -.05 | [-.11, .00] | -.05 | [-.11, .02] | -.04 | [-.11, .04] |
| Parks and recreation_(_*_t_*_-1)_ |  |  | .81 | [-.67, 2.29] | .80 | [-.62, 2.21] |
| Housing and community_(_*_t_*_-1)_ |  |  | .28 | [-.82, 1.37] | .23 | [-.91, 1.35] |
| Health_(_*_t_*_-1)_ |  |  | -.61 | [-1.27, .05] | **-.65** | **[-1.25, -.05]** |
| Hospitals_(_*_t_*_-1)_ |  |  | -.03 | [-22, .17] | -.03 | [-.21, .15] |
| Total operational_(_*_t_*_-1)_ |  |  | .05 | [-.04, .14] | .05 | [-.03, .13] |
| Demographic and economic covariates | |  |  |  |  |  |
| Median household income ($10,000) | |  |  |  | -.73 | [-1.87, .42] |
| Percent black (10%) |  |  |  |  | -1.40 | [-3.17, .36] |
| Population change (10%) |  |  |  |  | .19 | [-.80, 1.17] |

*Note.* Estimates in bold are significant at *p* <.05. Period and county fixed effects are included in all models.

References

1. Boardman JD, Powers DA, Padilla YC, Hummer RA. Low birth weight, social factors, and developmental outcomes among children in the United States. Demography. 2002;39(2):353–368.

2. Wise PH. Confronting racial disparities in infant mortality: Reconciling science and politics. American Journal of Preventive Medicine. 1993;9(6 Suppl):7–16.

3. Mathews TJ, MacDorman MF. Infant mortality statistics from the 2009 period linked birth/infant death data set. National vital statistics reports. 2013;61(8):1–28.

4. Grembowski D, Bekemeier B, Conrad D, Kreuter W. Are local health department expenditures related to racial disparities in mortality? Soc Sci Med. 2010 Dec;71(12):2057–65.

5. Bekemeier B, Grembowski D, Yang YR, Herting JR. Local public health delivery of maternal child health services: Are specific activities associated with reductions in Black–White mortality disparities? Matern Child Health J. 2011 Apr 20;16(3):615–23.

6. Bernet PM, Gumus G, Vishwasrao S. Effectiveness of public health spending on infant mortality in Florida, 2001–2014. Social Science & Medicine. 2018 Aug 1;211:31–8.

7. Bekemeier B, Yang Y, Dunbar MD, Pantazis A, Grembowski DE. Targeted health department expenditures benefit birth outcomes at the county level. Am J Prev Med. 2014;46(6):569–577.
